# Using Sentinel-1 and GRACE satellite data to monitor the hydrological variations within the Tulare Basin, California

**DOI:** 10.1038/s41598-022-07650-1

**Published:** 2022-03-09

**Authors:** Donald W. Vasco, Kyra H Kim, Tom G. Farr, J. T. Reager, David Bekaert, Simran S. Sangha, Jonny Rutqvist, Hiroko K. Beaudoing

**Affiliations:** 1 Lawrence Berkeley National Laboratory, University of California, Berkeley, CA 94720 USA; 2 Jet Propulsion Laboratory California Institute of Technology, Pasadena, CA USA; 3 Retired, Jet Propulsion Laboratory California Institute of Technology, Pasadena, CA USA; 4 Hydrological Sciences Lab, NASA GSFC, Greenbelt, MD USA; 5 Earth System Science Interdisciplinary Center, University of Maryland, College Park, MD USA

**Keywords:** Geophysics, Hydrogeology, Environmental sciences, Hydrology, Solid Earth sciences

## Abstract

Subsidence induced by groundwater depletion is a grave problem in many regions around the world, leading to a permanent loss of groundwater storage within an aquifer and even producing structural damage at the Earth’s surface. California’s Tulare Basin is no exception, experiencing about a meter of subsidence between 2015 and 2020. However, understanding the relationship between changes in groundwater volumes and ground deformation has proven difficult. We employ surface displacement measurements from Interferometric Synthetic Aperture Radar (InSAR) and gravimetric estimates of terrestrial water storage from the Gravity Recovery and Climate Experiment (GRACE) satellite pair to characterize the hydrological dynamics within the Tulare basin. The removal of the long-term aquifer compaction from the InSAR time series reveals coherent short-term variations that correlate with hydrological features. For example, in the winter of 2018–2019 uplift is observed at the confluence of several rivers and streams that drain into the southeastern edge of the basin. These observations, combined with estimates of mass changes obtained from the orbiting GRACE satellites, form the basis for imaging the monthly spatial variations in water volumes. This approach facilitates the quick and effective synthesis of InSAR and gravimetric datasets and will aid efforts to improve our understanding and management of groundwater resources around the world.

## Introduction

The Tulare Basin is an indispensable groundwater source within the Central Valley Aquifer system, which provides drinking water for 6.5 million residents and supports an agribusiness critical for the entire nation^1^. However, subsidence induced by groundwater depletion, while causing issues such as permanent storage loss and infrastructural damage, has been difficult to quantify and predict^2^. The hydrodynamics of the Tulare basin are quite complicated and the important components of the geologic system are not completely characterized.

**Figure 1:**
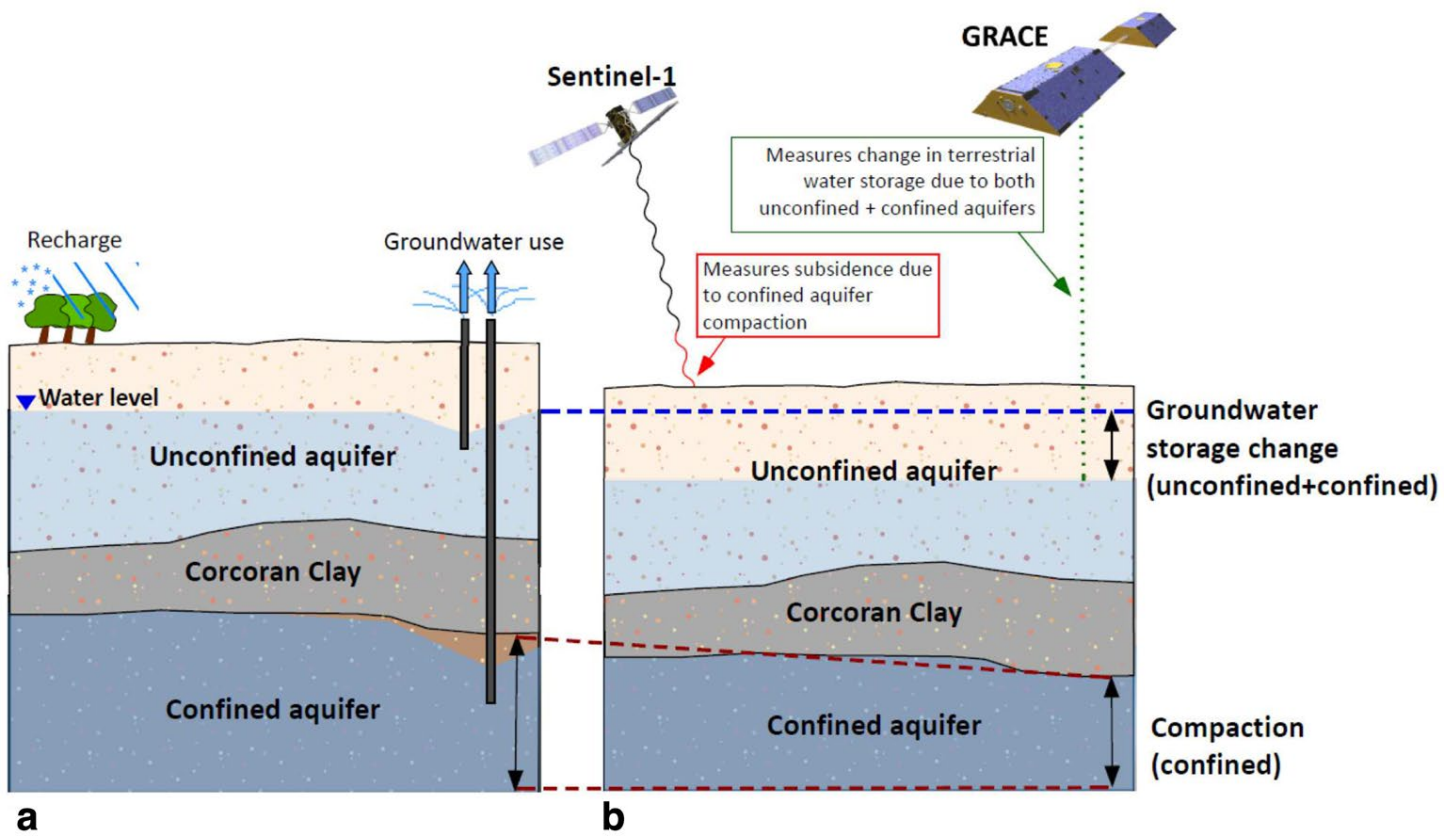
Schematic figure of the conceptual model of the Tulare basin aquifer. (**a**) The Corcoran clay separates the overlying unconfined aquifer from the confined aquifer below. Recharge occurs in the unconfined aquifer from snow, runoff, and precipitation. (**b**) Groundwater usage decreases overall terrestrial water storage in both the unconfined and confined regions (blue shaded region), which is detected by GRACE. Compaction (red dotted line), predominantly occurring in the confined aquifer, results in the line-of-sight displacement of the Earth’s surface measured by Sentinel-1 Synthetic Aperture Radar (SAR) satellites.

Furthermore, the complex hydrology of the basin, with multiple sources and sinks, can cause substantial changes over periods as short as a few months. Thus, orbiting satellite-based systems are well suited for monitoring variations within the Tulare basin at various timescales. Here, we consider Sentinel-1 Interferometric Synthetic Aperture Radar (InSAR) observations, which provide estimates of line-of-sight (LOS) displacements of the Earth’s surface, and terrestrial water storage (TWS) changes gravimetrically measured from NASA’s Gravity Recovery and Climate Experiment (GRACE) and GRACE Follow-on (FO) missions. Both data sets are sensitive to hydrologic variations in the Tulare basin and each has its own set of factors that complicate any analysis. For example, changes in the gravity field sensed by GRACE and GRACE-FO can be traced to a variety of sources such as ground movement, soil moisture, water table variations, and snow cover. Thus, it is difficult, if not impossible, to distinguish between water mass changes in the shallow unconfined aquifer and in the underlying confined aquifer using gravitational observations alone. Observations of surface deformation have their own issues, primarily due to the complicated relationship between ground motion and hydrological changes^3^. The main hydrological driver of deformation in a porous medium are typically changes in the total stress minus the fluid pressure within a given aquifer, a quantity known as the effective stress. In an unconfined aquifer, the fluid pressure is moderated by the possible upward movement of the water table and the coupling to the atmosphere, forming a constant pressure boundary condition. Ground deformation is often most strongly influenced by changes in the fluid volume in a confined aquifer, where the effective pressure can build up to large values. In addition, water volume changes in an overlying unconfined aquifer are coupled to the deeper aquifer, as it exerts a downward force upon the confining layer, leading to compressive stress and inducing further compaction. Thus, gravity and deformation data can have differing sensitivities to changes in the confined and unconfined aquifers and may be used together to distinguish changes in each. The presence of long-term inelastic deformation further complicates the interpretation of surface deformation, at it is related to earlier fluid volume changes and not to current aquifer conditions^4,5^.

In this paper, we describe an approach for removing longer-term deformation and extracting monthly variations in surface deformation. These shorter-term variations provide insight into the seasonal factors influencing the aquifer and its deformation. Combining the monthly displacement data with GRACE estimates of mass changes, we develop an inverse problem for water volume changes in a simplified model of the Tulare basin consisting of an unconfined near surface aquifer and an underlying confined aquifer (Fig. 1). We show that it is possible to fit both the GRACE and Sentinel observations with this simplified model, despite notable differences in the patterns of InSAR line-of-sight displacement and the gravitational mass changes. Furthermore, a comparison between water levels at wells with nearby geodetic observations indicates that the ground surface can move in both synchrony and in opposition with changes in the water table. This behavior highlights the complexity of the relationship between surface deformation and changes in the volume of water in the two aquifers.

## Results

### Interferometric synthetic aperture radar analysis

**Figure 2:**
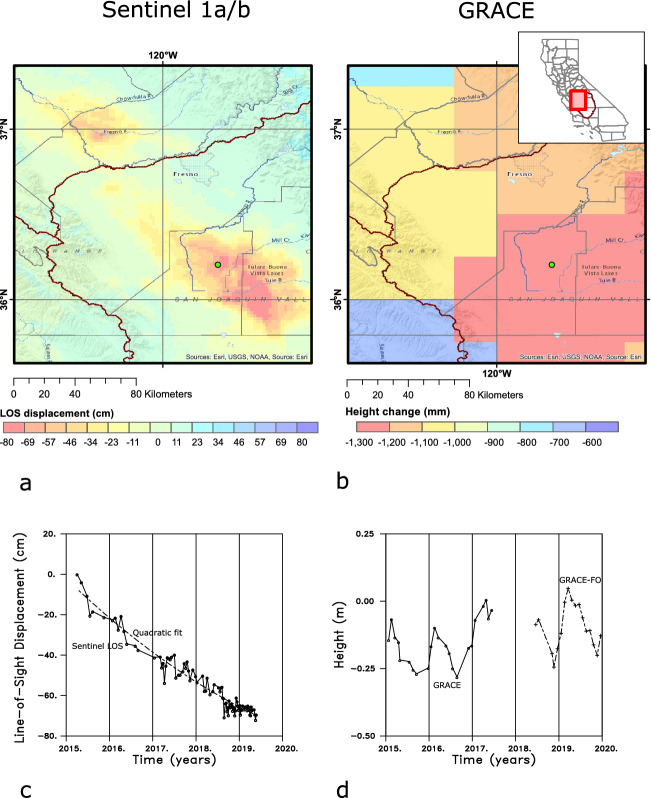
(**a**) Sentinel 1a/b Interferometric Synthetic Aperture Radar (InSAR) cumulative line-of-sight displacement from May 2015 until January 2019, for the area surrounding the Tulare basin. The filled green circle denotes the location used to calculate the InSAR line-of-sight displacements in panel c and in Fig. 3. (**b**) GRACE estimates of mass concentrations for 0.25 by 0.25 degree patches in the Tulare basin region, calculated in terms of an equivalent change in water height for the period 2011 to 2019. (**c**) Line-of-sight displacement time series for a point located midway between Lemoore and Corcoran. The solid curve and open circles denote displacements relative to early 2015 while the dashed curve represents a quadratic fit to the time series. (**d**) Area-wide average change in equivalent water height from 2015 until 2020 for the original GRACE satellite (solid line, open circles) and the subsequent GRACE follow-on (GRACE-FO) mission (dashed line, pluses). The average corresponds to the total change in water height over the mascons in the area of interest divided by the number of mascons in the region. The two maps in panels (**a**) and (**b**) were created with ESRI ArcMap 10.8.1 software (https://www.esri.com/).

Satellite-based Interferometric Synthetic Aperture Radar (InSAR) is currently the most widely used technique for monitoring surface deformation associated with groundwater variations and subsidence in the California Central Valley^4,6–11^. In this technique, phase shifts between radar returns gathered during successive passes of an orbiting satellite are used to estimate changes in the range or the line-of-sight (LOS) displacement^12^. Our estimates of LOS displacement were obtained from the Sentinel radar returns using the small baseline subset (SBAS) method^6,13–15^. The observed displacements are dominated by long term subsidence associated with the excessive pumping of groundwater from the Tulare basin^4,5^ (Fig. 2a,c). Previously, this trend has been removed by fitting linear and sinusoidal variations, as well as principal component analysis, and have somewhat successfully revealed secular and seasonal changes^4,5,11^. We adopt an alternative approach and fit a quadratic polynomial to each LOS displacement time series to remove the most significant long-term deformation (Fig. 2c).

**Figure 3:**
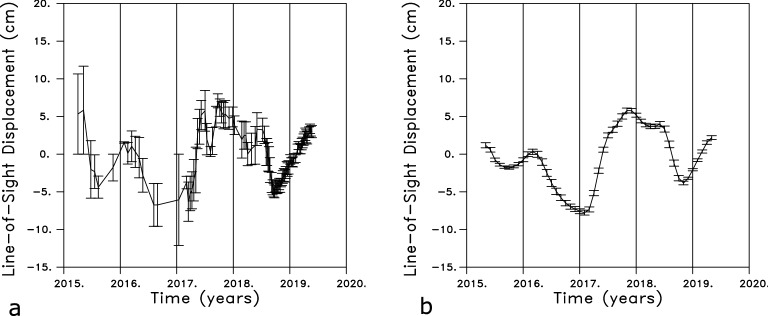
(**a**) Mean values for the reduced line-of-sight displacement with the quadratic fit removed [see Fig. 2c]. The values were obtained by averaging over a sliding three month window. The time series corresponds to the line-of-sight displacement for a point mid-way between Lemoore and Corcoran. (**b**) Time series obtained after averaging over 2 km by 2 km spatial bins. The error bars represent the one standard error about the mean value, obtained from the individual contributions to the bin average. The estimates are for the point denoted by the filled green circle in Fig. 2.

A three-month moving window was used to compute mean displacements and standard errors for each time series, as shown in Fig. 3a for a point located between the towns of Lemoore and Corcoran. The data was averaged in 2 km by 2 km spatial bins over an area of 180 km (east-west) by 220 km (north-south) to improve the signal-to-noise ratio and to provide estimates of mean line-of-sight displacement values and their standard errors. This averaging significantly smoothed the data and further reduced the standard deviations associated with the estimated mean values in each time-space window (Fig. 3b). The size of the bins was chosen in order to have at least 20 measurements for each estimate of the mean value, and to be closer to the 6–7 kilometer-scale of the interpolated GRACE gravity data than are the original LOS estimates. The resulting 9900 time series were re-interpolated onto monthly displacements. Observations from Global Positioning System stations in and around the Central Valley have been shown to be sensitive to hydrological variations in the region^11,16^. Two time series, for locations corresponding to the Global Positioning System stations LEMA (near the town of Lemoore) and CRCN (near the town of Corcoran), are shown in the Supplementary Figure S1, along with the daily changes obtained from the GPS observations. The InSAR LOS displacements are with respect to a reference point that is assumed to be stationary, while the GPS estimates are with respect to a reference datum such as the North American plate. After accounting for this difference we find general agreement between the InSAR and GPS estimates of line-of-sight displacement at the two stations.

**Figure 4:**
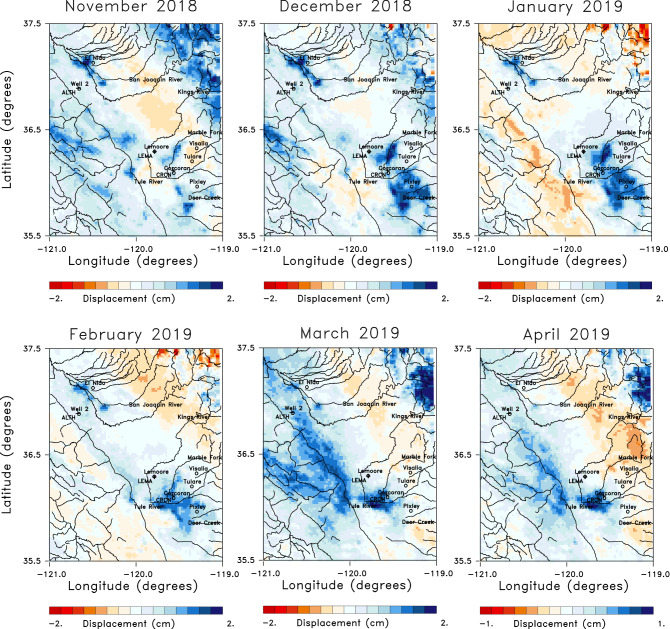
Six monthly line-of-sight displacements from the years 2018 and 2019. Rivers and streams in the region are indicated by the solid lines while several towns in the area are denoted by open circles and labeled. The locations of the GPS stations ALTH, LEMA, and CRCN are also shown as +’s in this panel. The location of a monitoring well discussed in this paper, water well 2, is denoted by an open circle. The colors represent the line-of-sight displacements obtained after removing the long term quadratic trend from each displacement time series. The plots were constructed using NCL and NCAR Graphics 6.5.0 (https://www.earthsystemgrid.org/dataset/ncl.650.html).

The Sentinel-1 mission had a repeat time of 12 to 24 days through the entire observational period, and the derived line-of-sight displacement is resolved at a spatial resolution of 90 m^6^. As is clearly seen in Figs. 2c and 3, the time sampling between late 2016 and mid-2018 is somewhat irregular with clear gaps, particularly in late 2016, perhaps due to a loss of coherence in certain agricultural areas. At JPL’s request, the satellite repeat time was reduced to 6 days from about mid-2018 onward, resulting in higher quality monthly estimates for this later time period. Thus, we analyzed monthly changes during this better-sampled interval. In Fig. 4, we plot in map view the six monthly changes from November 2018 through April 2019. There is notable uplift in the southeast quadrant from December 2018 through February 2019, and in a narrow southeasterly oriented zone to the northwest. The uplift is in regions where rivers draining the Sierra Nevada enter the Central Valley^11^. This uplift spreads laterally in February, March, and April, joining to form a larger northwestern region trend along the deeper Tulare basin. Interestingly, the trend of both the GPS and InSAR LOS displacements are positive throughout 2017 (Fig. S1). These increases stand in contrast to the significant downward slopes observed in the years 2015, 2016, and 2018.

**Figure 5:**
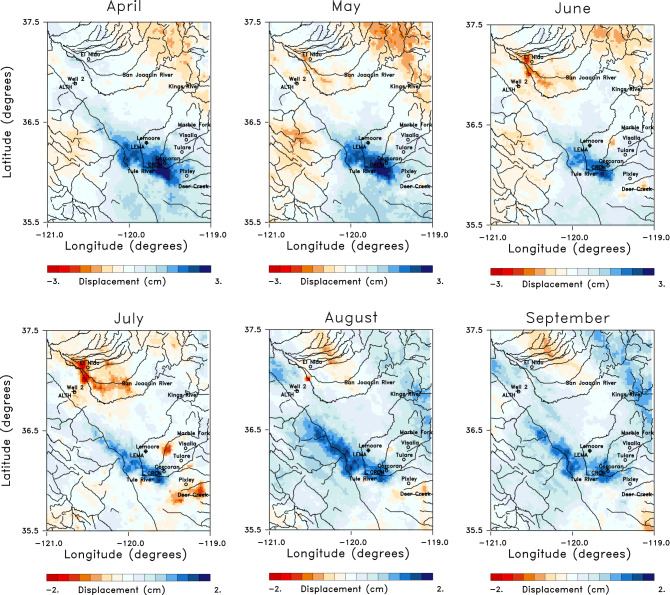
Panels displaying 6 sequential instances of monthly line-of-sight displacements for months in 2017. The plots were constructed using NCL and NCAR Graphics 6.5.0 (https://www.earthsystemgrid.org/dataset/ncl.650.html).

**Figure 6:**
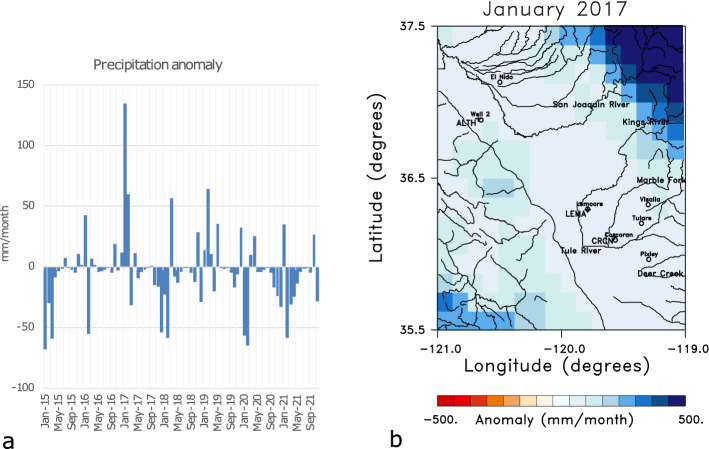
Precipitation data used as a forcing function for Phase 2 of the North American Land Data Assimilation System (NLDAS-2)^17^. The precipitation data extends from 1979 to the present at a spatial resolution of 0.125 degree and the monthly precipitation fields are accessible from the NASA Goddard Earth Science Data and Information Services Center [see NLDAS_FORA0125_M.002 doi:10.5067/Z62LT6J96R4F]. (**a**) Precipitation anomaly time series for the entire study area from the beginning of 2015 to the end of 2021. The anomaly is in millimeters per month. (b) Map of the precipitation anomaly for the area for the excessively wet month of January 2017. The map in panel (**b**) was constructed using NCL and NCAR Graphics 6.5.0 (https://www.earthsystemgrid.org/dataset/ncl.650.html).

Though we will only analyze a subset of the LOS estimates shown in Fig. 4, it is important to look at other time intervals in order to understand the yearly variations in the region. To this end, in Fig. 5 we display the displacements from the relatively wet year 2017. The six monthly changes shown in the Figure, from April to September 2017, display interesting temporal variations. In April and May, there is significant uplift in the southern end of the basin, most likely due to the unusually large rainfall in late 2016 and early 2017 that is evident in the precipitation anomaly time series plotted in Fig. 6a.

This is followed by two months of reduced uplift and even subsidence in some areas of the Tulare basin in June and July, though the region of the largest uplift in April and May is still rising. The area of uplift parallels that observed for April of 2019 and plotted in Fig. 4. There is also an increase in uplift through August and September 2019, which was initially surprising to us, given that these were dry months for the region. However, an examination of stream and river flows into the region (Supplementary Fig. S2) suggests this later uplift is due to the effects of the runoff from large accumulations of snowmelt at higher elevations. As shown in the river discharge data in Fig. S2, the snowmelt leads to a secondary influx of water in mid to late summer of 2017, particularly in rivers draining mountainous areas, such as the Marble Fork river. The increased water volume at lower elevations appears to have given rise to higher fluid pressure in the confined aquifers of the Tulare basin and subsequent expansion of the confined aquifer beneath the Corcoran clay and the overlying formations. The 2017 LOS displacements in Fig. 5 are associated with the high levels of rain- and snow-fall in late 2016 and early 2017, as indicated in Fig. 6a. The area with the highest levels of precipitation in January 2017 is in the Sierra Nevada to the east of the Tulare basin (Fig. 6b). Much of this precipitation represents accumulating snow, the source of the significant runoff in the summer months.

### GRACE gravity observations

While the InSAR line-of-sight displacements are likely to be the most sensitive to fluid pressure and corresponding effective stress changes within the confined aquifer, GRACE gravity observations are influenced by water mass changes everywhere in the Tulare basin^18^. In particular, it is not possible to distinguish between changes in the shallow unconfined aquifer and the deeper confined aquifer with satellite-based gravity data. There have been several discussions and comparisons of InSAR and GPS data to GRACE estimates of mass variations over time^9,19–22^. The two panels Fig. 2b,d highlight the limitations of the GRACE observations obtained during the interval of interest, from 2015 to mid-2019. Figure 2b presents the changes in mass estimated by the GRACE in the manner that they are obtained from the University of Texas Center for Space Research, as equivalent changes in water height. The 1/4th of a degree GRACE estimates of mass concentrations (mascons) that we use are of much lower resolution than the InSAR observations. In particular, the spacing between mascons is roughly 28 km, compared to the 2 km by 2 km bins used for displacement estimates. Furthermore, the physical resolution is actually much less-around 1 degree by 1 degree at the equator^23–26^- leading to the large-scale anomalies in Fig. 2. In addition, the temporal sampling is somewhat irregular and there is a notable gap from June 2017 until June 2018 (see Fig. 2d) due to the transition from the original GRACE satellites to the GRACE-FO (follow-on) satellites^27^. Thus, the wet year of 2017 is not well sampled and we must look at a later time, such as after June 2018, in order to conduct a joint inversion.

**Figure 7:**
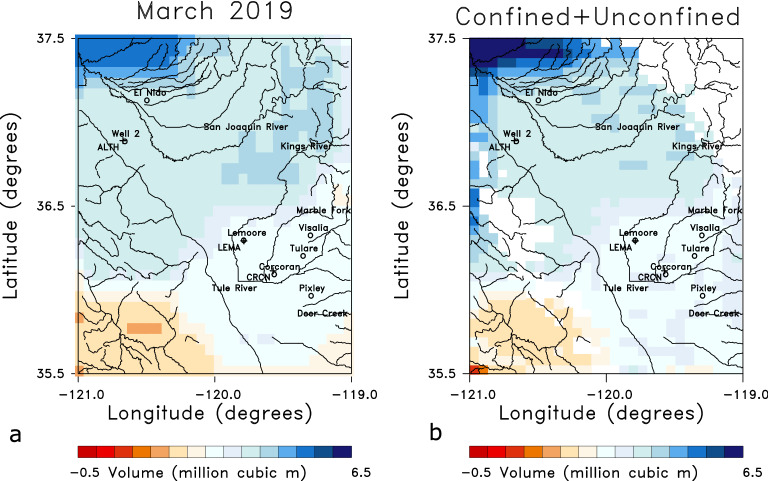
(**a**) GRACE 1/4th degree mascons corresponding to changes in March 2019, which have been sub-divided into 4x4 sub-grids and re-interpolated onto a finer grid that correlates with the Sentinel InSAR estimates. The color scale indicates the volume changes in millions of cubic meters during the month of March 2019. The water volume change has been reduced to reflect the smaller area (roughly 6km by 6km) that is represented in the finer grid. (**b**) The sum of the water volume changes in the unconfined and confined aquifers of the inversion result that is plotted in Fig. 8. The open circles denote towns in the area while the +’s indicate the locations of three GPS stations. The solid curves indicate rivers and streams in the region. The plots were constructed using NCL and NCAR Graphics 6.5.0 (https://www.earthsystemgrid.org/dataset/ncl.650.html).

An example of current GRACE estimates, corresponding to mass changes during March in 2019, are plotted in Fig. 7a. As noted above, this later time interval was chosen because of the higher quality InSAR displacement estimates post-2018 and the availability of the GRACE-FO observations starting in mid-2018. Note that we have sub-divided each mascon into 4 smaller pixels with dimensions of roughly 6 by 6 km, and the mass was divided by 1/16th, in order to maintain a spatial scale that is consistent with our interpretation of the Sentinel InSAR data. The mass concentrations were converted to water volume changes in order to conduct a uniform analysis of the GRACE and InSAR data. To focus on shorter-term monthly changes, the long-term trend of the GRACE total water storage (TWS) was removed from each time series by fitting a quadratic curve to the values between January 1, 2011 and January 1, 2020. Note the difference in the pattern of volume change as compared to the pattern of displacement in March 2019, plotted in Fig. 4. The ground surface is subsiding in much of the eastern half of the area and uplifting to the west in March 2019, while Fig. 7a indicates an overall increase in the water mass with the exception of a slight mass decrease in the southwest corner.

### A constrained inversion for water volume changes

We conducted a constrained inversion of the GRACE data, where the constraints are provided by InSAR estimates of volume change in the confined aquifer. The details of the inversion are presented in the Methods section below, but the model consists of two volumes representing the shallower unconfined aquifer and the underlying confined aquifer, with the Corcoran clay defining the boundary between the two^28^ (Fig. 1). Within the model, this boundary was extended beyond the extent of the Corcoran clay to allow for an effective confined aquifer to the east of the clay layer. The surface deformation is hypothesized to be driven primarily by the movement of the boundary between the confined and unconfined aquifers, due to changes in the mass of overlying material or changes in effective stress within the confined aquifer. The inversion proceeds in two main steps: in the first step we use the InSAR displacements to solve for the individual volume changes in all of the *N* grid blocks of the confined aquifer, which we denote by $\delta V_n^{InSAR}$. In the next step we use the $N_g$ GRACE-derived gravity changes, $\delta g_l$, and the InSAR-derived confined aquifer volume changes to estimate the water volume changes in the unconfined ($\delta V_n^u$) and the confined aquifers ($\delta V_n^c$), given by the systems of equations (5) and (6) in the Methods section, which we repeat here for convenience

$$\begin{aligned} \delta V_n^{InSAR}= & {} - \frac{\rho g l_o}{K_u} \cdot \delta V_n^u + B \cdot \delta V_n^c\\ \delta g_l= & {} \sum _{n=1}^N G_{ln}^u \delta V_n^u + \sum _{n=1}^N G_{ln}^c \delta V_n^c , \end{aligned}$$

where $n = 1,2,\ldots ,N$ and $l=1,2,\ldots N_g$ for the $N_g$ GRACE estimates of gravity change. In these equations, $G_{ln}^u$ and $G_{ln}^c$ are the Green’s functions derived using expressions for the gravitational attraction due to a rectangular prism^29–32^, $\rho$ is the density of the groundwater, *g* is the gravitational constant, and $l_o$ is the vertical extent of the aquifer used to calculate the reference volume. The porous medium is characterized by the undrained Bulk modulus, $K_u$, and by Skempton’s coefficient *B*^33,34^. The parameters $K_u$ and *B* in the equations were determined by a systematic grid search in which the misfit was minimized, giving an undrained bulk modulus of 0.3 GPa and a Skempton’s coefficient of 0.97 which are compatible with earlier findings^8^.

**Figure 8:**
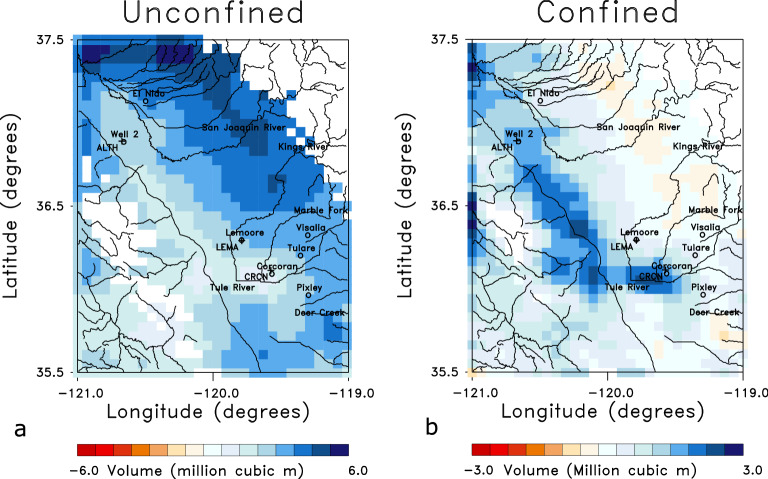
Estimates of the water volume changes in the unconfined (**a**) and confined (**b**) aquifers of the model. The color scale denotes the estimated water volume changes in millions of cubic kilometers during March 2019. Areas with elevations above 600 meters have been removed from the solution because they are likely to be in anomalous mountain areas that do not conform to the model assumptions. The labeling indicates towns, GPS stations, and rivers as denoted in the previous captions. The plots were constructed using NCL and NCAR Graphics 6.5.0 (https://www.earthsystemgrid.org/dataset/ncl.650.html).

The solution to the coupled linear equations given above are found using an iterative and regularized solver^35^. In Fig. 8 we plot the resulting estimates of water volume changes occurring in the unconfined and confined aquifers during the month of March in 2019. Areas with elevations exceeding 600 m were removed from the solutions as they are likely to be adversely influenced by snow and have groundwater hydrology that is significantly different from the Central Valley sediments (white regions in Fig. 8). In the unconfined aquifer, there are large volume increases at the western edge of the Sierra Nevada and the southern edge of the basin where the rivers and streams most likely contribute significant water volumes. The solution for the volume changes in the confined aquifer does resemble the observed InSAR displacements plotted in Fig. 4, albeit with some deviations in the north-western corner where higher volume increases are required to fit the gravity data.

The sum of the volume changes in the two layers, plotted in Fig. 7b, is in fairly good agreement with the GRACE mascon estimates of equivalent water volume change (Fig. 7a). A more quantitative comparison between the reference (GRACE-derived) gravity changes and gravity changes calculated using the volume changes from the inversion is plotted in the Supplementary Fig. S3. In addition, in Fig. S3 we plot the normalized left-hand-sides (Observed) and right-hand-sides (Calculated) of the InSAR constraint provided by the first set of equations given above. Both sets of equations are satisfied by the model shown in Fig. 8. The largest misfits for the gravity data are associated with observations at the edge of the model where mass changes outside the area of interest can influence the values. Thus, it appears possible to honor both the Sentinel InSAR and the GRACE gravity data with a simple model involving a confined and an unconfined aquifer. By looking at shorter-term monthly changes we are minimizing the impact of poorly known parameters, such as the inelastic skeletal storage properties which influence longer-term behavior.

## Discussion

Our analysis of the Sentinel InSAR and GRACE gravity data is relatively straight-forward and involves several simple steps, such as removing a long-term quadratic trend and averaging in both time and space. The two-volume aquifer model, consisting of unconfined and confined aquifers, satisfies both the Sentinel and GRACE constraints, suggesting that the datasets may be explained by a common hydrological source. For the particular month that we considered in detail, March 2019, there is a volume increase within the overlying unconfined aquifer at the eastern edge of the Central Valley (Fig. 8a), perhaps due to a combination of preceding winter rains and the early onset of snowmelt. In the confined aquifer of the model (Fig. 8b), the region in the Central Valley is dominated by a northwest oriented volume increase that follows the deeper region of the aquifer. The changes in Fig. 4 suggest that the source of this volume increase is due to the influx of water from rivers primarily in the southern Sierra Nevada and in an area to the north. The resulting pattern of uplift in March and April of 2019 shares many characteristics to the changes in April 2017 (Fig. 5), suggesting similar seasonal variations.

**Figure 9:**
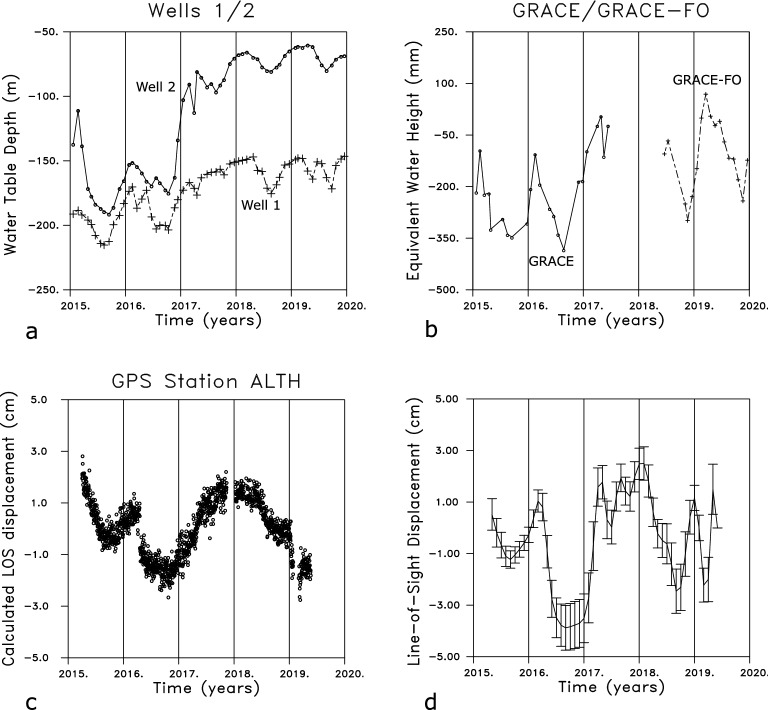
(**a**) Water table variations at well 1 (365322120401203) and well 2 (365325120391504) that are within a few hundred meters of each other. The continuous water table observations are available from the site: https://data.cnra.ca.gov/dataset/continuous-groundwater-level-measurements The locations of the wells are indicated in Fig. 4 by an open circle and the label well 2. (**b**) Equivalent water height changes for a point corresponding to well 1 obtained from the GRACE and Grace follow-on (GRACE-FO) missions. The symbols denote the sampled values and the gap corresponds to the transition between the two missions when no observations were available. (**c**) Vertical displacements from the GPS station ALTH near the two water wells 1 and 2. The open circles indicate values from the daily recordings. (d) Estimated line-of-sight displacements at the location of water well 1.

While very few wells have monthly observations of the water table in this area, we did find two closely-spaced and densely-sampled monitoring wells in the northwest portion of our study area (Fig.  9). Both wells display large long-period seasonal trends with a period of about 1 year. The periodic seasonal variation is interrupted by a systematic increase in water level from late 2016 to early 2018 due to the excessively wet winter of 2017, which is largely reflected in the GRACE trends in Fig. 9b. Unfortunately, the break in the satellite coverage in 2017 and 2018 means that it is not possible to determine if the gravitational signal from the water volume continued to build up in 2017 before falling in 2018, as observed in the geodetic data. The vertical displacements recorded at a nearby GPS station ALTH records uplift during all of 2017, in correspondence with the upward movement of the water table, followed by a systematic decrease in 2018. This pattern is also seen in the InSAR LOS data extracted for the same location (Fig. 9d). Note that the ground displacements and the water levels diverge in 2018 and 2019, when the water level remains elevated, while the ground surface subsides, as observed in both the GPS and InSAR displacements in Fig. S1. In addition, the water table in Wells 1 and 2 appear stable during the early months of 2019, while the ground deformation indicates early subsidence in January and February followed by uplift in March and April of 2019, supporting the notion of a stable water table during deformation driven by the confined aquifer.

**Figure 10:**
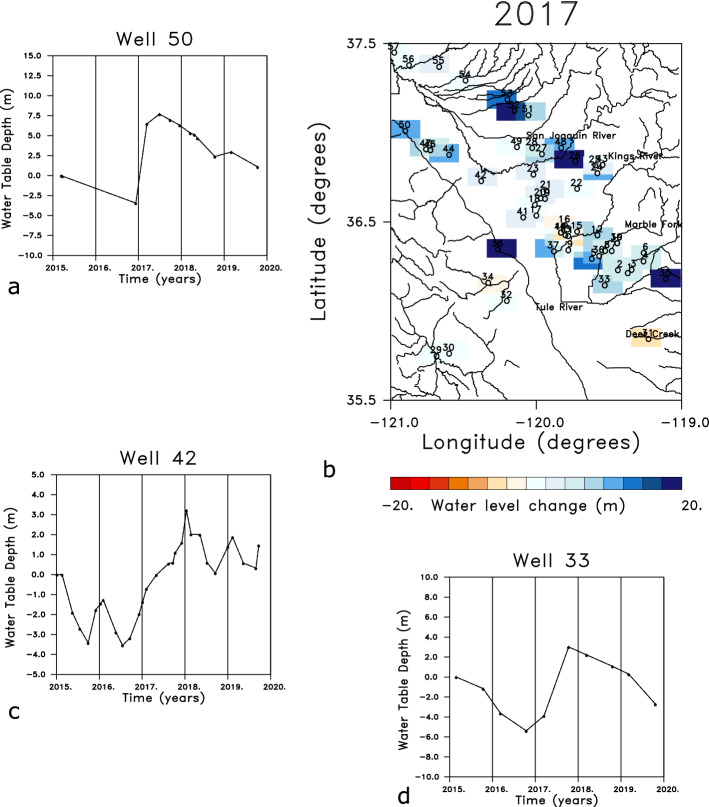
(**a**) Water level changes observed in Well 50, denoted in the location map in panel b. The time series has been reduced by shifting the initial value to zero and removing a linear trend from the data. (**b**) Location map indicating the position of the 57 wells that had at least 10 observations between the start of 2015 and the end of 2020. The colored rectangles denote the changes in the water levels in each well that occurred during the water year 2017 (between October 2016 and October 2017). (**c**) Water level changes in Well 42 obtained after shifting the curve such that the initial value is zero and removing a linear trend. (**d**) Changes in the depth to the water table in Well 33, reduced in the same fashion as the other two wells in this figure. The continuous water table observations are available from the site: https://data.cnra.ca.gov/dataset/continuous-groundwater-level-measurements while the seasonal data may be found at https://data.cnra.ca.gov/dataset/periodic-groundwater-level-measurements. The plots were constructed using NCL and NCAR Graphics 6.5.0 (https://www.earthsystemgrid.org/dataset/ncl.650.html).

The long term behavior of the water table in the region is constrained by an additional 57 wells that are sampled roughly twice a year, as shown in Fig. 10. The time series for three widely-spaced wells, displaying changes in the water table between 2015 and 2020, somewhat mirrors the behavior of the two wells plotted in Fig. 9. In particular, there is a sustained elevation of the water table from the end of 2016 until some time in late 2017 and early 2018. The wide-spread nature of this change is evident in the regional map in Fig. 10b, indicating the change in the water table for the water year 2017, that is from October 2016 to October 2017. Almost all of the available observation wells record upward movement in the water table of 5–10 m. In the time series in Fig. 10 we observe a rapid build up in early 2017 and a gradual decline in 2018 and 2019. The rapid decay observed in the GPS and InSAR observations in Fig. 9 and Supplementary Fig. S1 is not seen in the water table changes in wells 33 and 50. Thus, it appears that the water level and the ground surface can move in synchrony, due to water volume increases in the unconfined and confined aquifers, and in opposition, due to groundwater loading of the confined aquifer in conjunction with deep groundwater withdrawal.

More work is necessary to substantiate and fully understand these results, and to determine the most important contributions to ground deformation. For example, continued monitoring is needed in order to determine if the patterns observed around April of 2017 and 2019 are truly periodic seasonal changes driven by the groundwater hydrology. Detailed modeling of the flow and the propagation of subsurface fluid pressure changes will help in understanding the dynamics of these results and other observations^11^, and to replicate these observations. A larger scale study will be better suited to the resolution of the GRACE data and will allow for a more comprehensive comparison with observations of water levels in monitoring wells. Improved characterization utilizing archived well logs and borehole extensometer data is necessary in order to develop a better geomechanical model of the system and to obtain better estimates of poroelastic properties. It is particularly important to determine the relationship between effective stress changes in the confined aquifer and the resulting volume changes. Still, the results here do suggest that available Sentinel and GRACE satellite data can indeed monitor hydrological variations over time scales of a month or more. With future improvements in observations, such as the NASA-ISRO SAR (NISAR) mission planned for 2023, there should be even better constraints on temporal changes in the Tulare basin in the future. Longer wavelength L-band data, such as the ALOS-PALSAR observations^8,36^ can improve imaging in highly vegetated regions but they did not have sufficient temporal resolution for this study.

## Methods

Our analysis is based upon the simplified model of the Tulare basin outlined in Fig. 1, consisting of a shallow aquifer from the water table down to a mostly impermeable but deformable boundary, which for much of the region is defined by the Corcoran clay^28^. However, due to factors such as layering, it is frequently true that the vertical permeability is an order of magnitude less than the horizontal permeability so that other parts of the basin may contain partially confined aquifers, particularly over short time intervals. The underlying sequence of layers collectively forms a confined aquifer and effective stress changes within this volume lead to changes in the vertical location of any overlying deformable boundaries, such as the ground surface. The upper boundary of the confined aquifer is subject to a downward force, due to the weight of the overlying sediments and water, including soil moisture and snow, and sediment volumes. It is also subject to any changes in effective stress within the confined aquifer itself. The relationship between a change in the confining pressure $dP_c$, the total volumetric stress, and the changes in the volume of the solid and water volumes, $dV_s^c$ and $dV_w^c$ respectively, in the confined aquifer is^34,37^

1 $$\begin{aligned} \frac{1}{K_u} dP_c = - \frac{dV_s^c}{V_o} + B \frac{dV_w^c}{V_o}, \end{aligned}$$

assuming poroelastic behavior for the monthly changes, where $K_u$ is the effective undrained bulk modulus of the sediments comprising the confined aquifer at this location and *B* is Skempton’s coefficient^33,34^. We will assume that the mass of the overlying solid material is constant and that only the overlying water volume is changing, so that

2 $$\begin{aligned} dP_c = \rho g \cdot dh = \rho g \frac{dV_w^u}{A_o} \end{aligned}$$

where $\rho$ is the density of the water, *g* is the gravitational constant, and $A_o$ is the horizontal surface area of the top of the grid block. Substituting equation (2) into the first equation produces an expression relating the change in the volume of water overlying the grid block block to the solid and water volume changes within the grid block of the confined aquifer. We can rearrange this equation and multiply by the reference volume of the grid block $V_o$, solving for the solid volume change in terms of the water volume changes in the unconfined and confined aquifers

3 $$\begin{aligned} dV_s^c = - \frac{\rho g l_o}{K_u} \cdot dV_w^u + B \cdot dV_w^c , \end{aligned}$$

where $l_o$ is the vertical extent of the aquifer at the corresponding location used in the calculation of the reference volume.

We can estimate the solid volume changes $dV_s^c$ from the InSAR line-of-sight changes using the inversion methods developed for geodetic data^10,38–40^. A finite incremental change in solid volume for the i-th grid block, obtained from the InSAR observations, is noted by $\delta V_i^{InSAR}$. Assuming that the medium overlying the confined aquifer behaves as an elastic medium during the time increment of interest, typically 6 to 11 days, the inverse problem involves solving the linear system for the solid volume changes for each grid block in the confined aquifer

4 $$\begin{aligned} \delta l_i = \sum _{n=1}^{N} U_{in} \delta V_n^{InSAR} \end{aligned}$$

where $\delta l_i$ is the i-th InSAR line-of-sight observation and $U_{in}$ is a discrete version of the Green’s function relating aquifer volume change to the line-of-sight displacement of the Earth’s surface. Using the InSAR estimates of volume change as a constraint, forming the left-hand-side of equation (3) we can write down an InSAR-based constraint defined by the force balance across the confining layer, for each of the N grid blocks of the two layers

5 $$\begin{aligned} \delta V_n^{InSAR} = - \frac{\rho g l_o}{K_u} \cdot \delta V_n^u + B \cdot \delta V_n^c , \end{aligned}$$

for $$n = 1,2,...,N$$.

In addition, we have the constraint due to the mascons obtained from the analysis of the GRACE data. It is not straight-forward to relate volume or mass changes at the Earth’s surface to confined and unconfined aquifer water volume changes. Furthermore, the edges of the mascons are artificial boundaries introduced in the formulation of the inverse problem that maps the GRACE data into changes in mass at the Earth’s surface^26^. To mitigate these issues we use the mascons to generate gravitational changes at a height above the Earth’s surface. We use a height of 6000 meters as that is the lateral dimensions of our grid blocks for the inversion. An additional increase in elevation will also increase the sensitivity of the gravity values to changes that are outside of the Tulare basin. Thus, we solve a forward problem and calculate the gravity changes at a height of 6000 meters and then use these changes as data for an inverse problem for water volume changes in the confined and unconfined aquifers

6 $$\begin{aligned} \delta g_l = \sum _{n=1}^N G_{ln}^u \delta V_n^u + \sum _{n=1}^N G_{ln}^c \delta V_n^c \end{aligned}$$

with $l=1,2,...N_g$ for $N_g$ gravity estimates, and where $G_{ln}^u$ and $G_{ln}^c$ are the Green’s functions for the gravitational attraction of a rectangular prism^29–31^. Such Green’s functions have proven useful in the analysis of airborne gravity and gravity gradiometry data^32^. The inverse problem for the water volume changes, compatible with the InSAR volume change estimates, involves solving the linear system defined by equations (5) and (6).

## Supplementary Information

Supplementary Information.

## Data Availability

The InSAR observations discussed in the paper were originally provided by Caltech’s Jet Propulsion Laboratory (JPL), ARIA project and are available as an ARIA product from JPL or from our Zenodo archive, the citation is: Donald Vasco (2019). Central Valley InSAR Data - 2015-2016-2017-2018 (Version 1.0), DOI 10.5281/Zenodo.3468553. As noted above, the GRACE data are freely available from the University of Texas Center for Space Research as CSR-GRACE-GRACE-FO-RL06-Mascons-all-corrections-v02.nc.
