# Supplementary material for: Using Sentinel-1 and GRACE satellite data to monitor the hydrological variations within the Tulare Basin, California
Source: Sci Rep. 2022 Mar 9;12:3867. doi: 10.1038/s41598-022-07650-1 (PMC8907331; doi:10.1038/s41598-022-07650-1)
Supplement: Supplementary file 1 — Supplementary Information. [file 41598_2022_7650_MOESM1_ESM.pdf]

Supplementary Figures

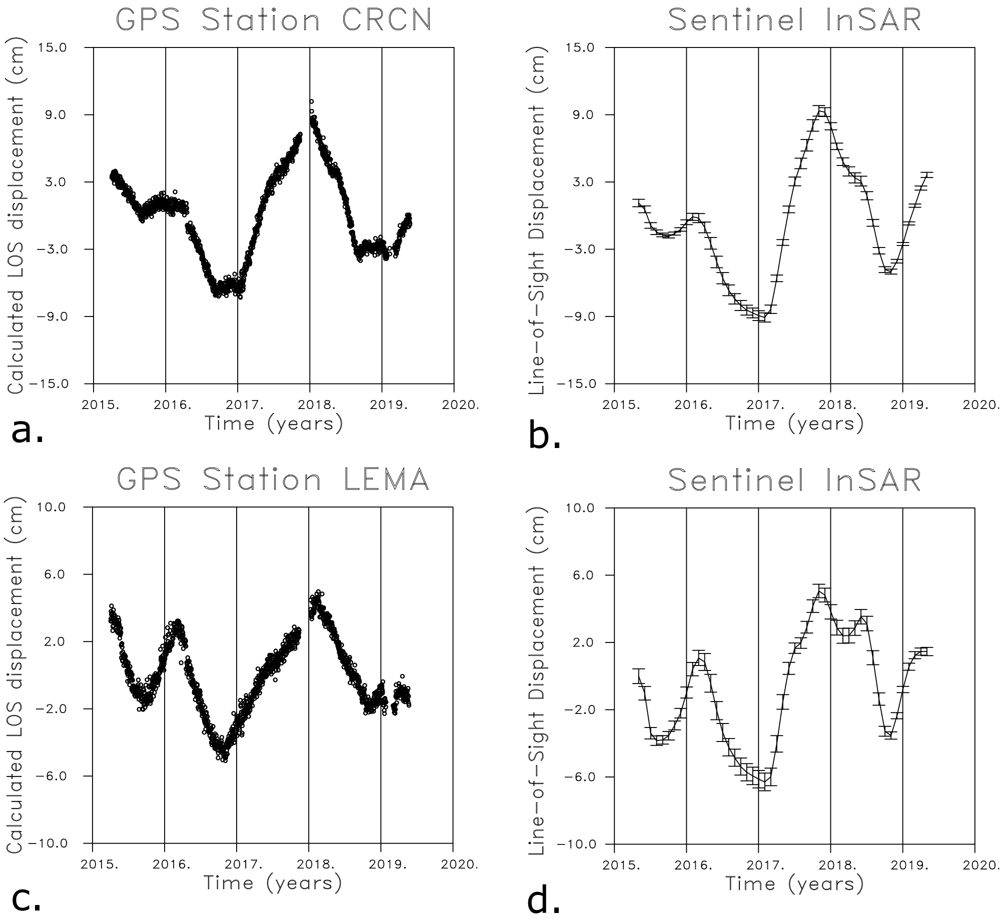

Supplementary Figure S 1: (a) Reduced estimate of the line-of-sight displacement at the GPS station CRCN near the center of the large uplift in Figure S4. (b) Reduced line-of-sight displacement at the point nearest to the Global Positioning System (GPS) station CRCN. (c) Reduced estimate of the line-of-sight displacement at GPS station LEMA. (d) Reduced Sentinel InSAR line-of-sight displacement at the location of station LEMA. The Global Navigation Satellite System (GNSS) observations, in this case from the U.S. GPS network have been projected onto the satellite look vector in order to compare line-of-sight displacements. Note that there may be an arbitrary offset in displacement due to possible variations between the reference point of the InSAR estimates and the datum of the GPS displacements.

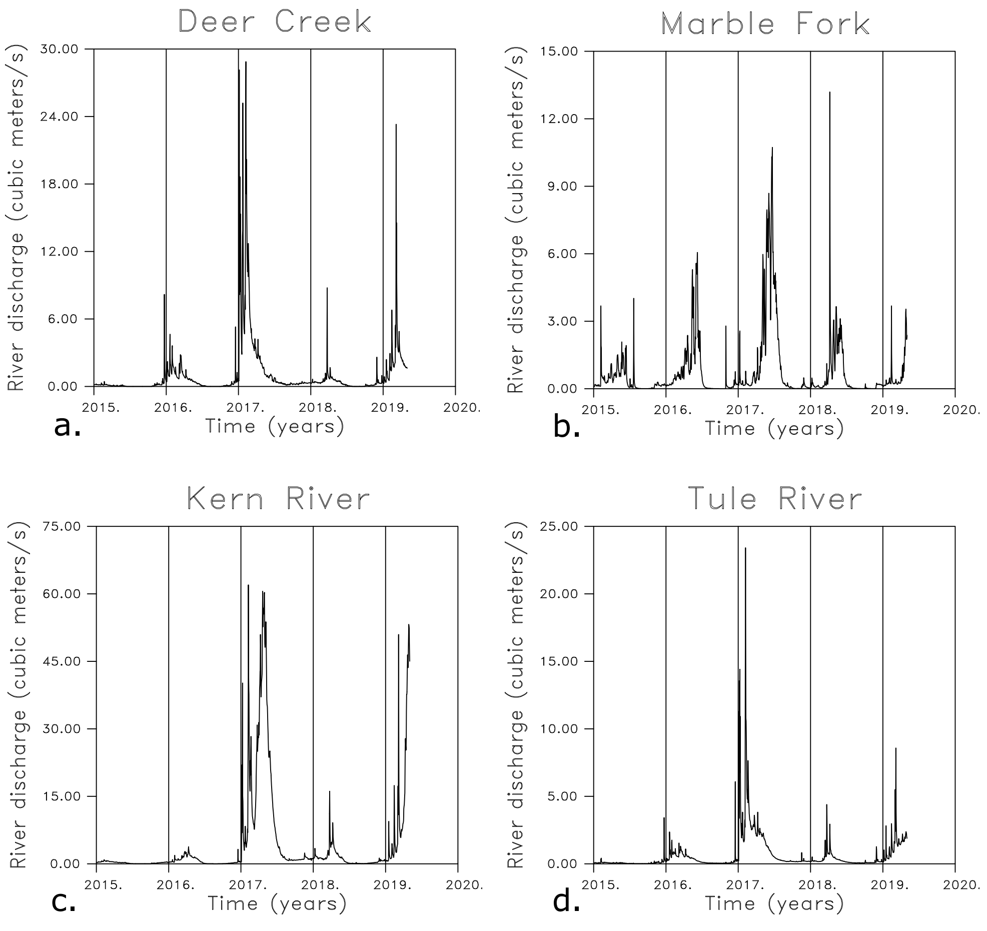

Supplementary Figure S 2: River discharge data for four rivers that drain into the Tulare basin, obtained from the U.S. Geological Survey [https://waterdata.usgs.gov/nwis]. Three of these rivers are plotted on the maps of the area of interest. The Kern River, which drains into the southern portion of the region, lies just to the south of our study area.

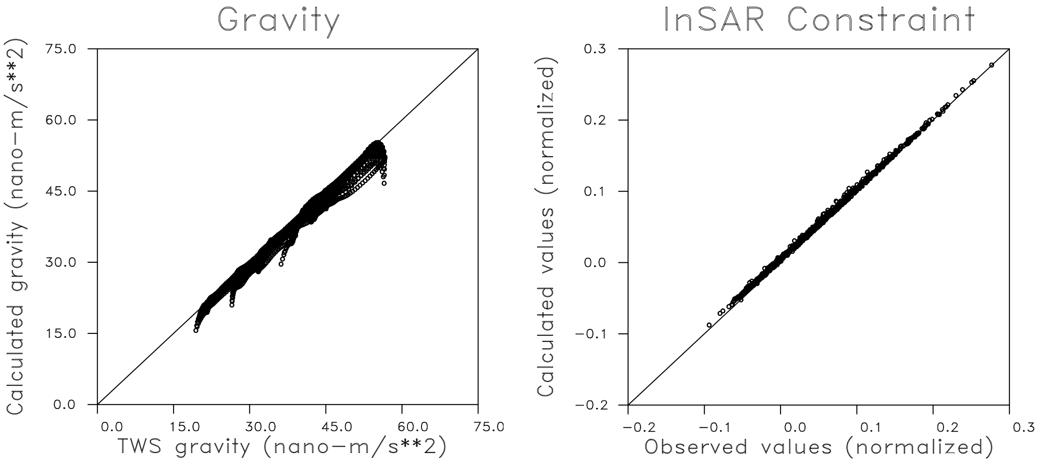

Supplementary Figure S 3: (a.) Observed gravity changes at an elevation of 6000 meters over the region plotted against the values predicted using the water volume changes from inversion result shown in Figure 4. (b.) Fit to the InSAR constraint given by equation (5).
